# Gender gap–Gender-specific development in the field of obstetrics and gynecology in Germany in the last 20 years

**DOI:** 10.3389/fmed.2023.1207388

**Published:** 2023-12-07

**Authors:** Stefan Hertling, Mario Kaiser, Ekkehard Schleußner, Franziska Maria Loos, Niklas Eckhardt, Isabel Graul

**Affiliations:** ^1^Department of Obstetrics and Gynecology, University Hospital Jena, Jena, Germany; ^2^Department of Orthopedic, Campus Eisenberg, University Hospital Jena, Eisenberg, Germany; ^3^Jenoptik GmbH, Jena, Germany; ^4^Practice for Orthopedics and Shoulder Surgery Leipzig, Leipzig, Germany; ^5^Institute for Diagnostic and Interventional Radiology, University Hospital Jena, Jena, Germany; ^6^Department of Trauma-, Hand and Reconstructive Surgery, University of Jena, Jena, Germany

**Keywords:** gender gap, gynecology, obstetrics, Germany, women, leadership

## Abstract

**Background:**

Gender Gap refers to differences between men and women in terms of access to medical education, career development, and leadership positions in medical practice and research. Although women now make up most medical school graduates in many countries, they are often underrepresented in higher positions.

**Objective:**

The aim of this study is therefore to analyze the gender-specific development in the field of Obstetrics and Gynecology in Germany over the past 20 years and to survey the current *status quo*.

**Materials and methods:**

An narrative review was carried out on the development of female graduates of human medicine, the proportion of women in contract medical care and clinical care, as well as the gender-specific evaluation of obtaining a gynecological/obstetric additional qualification. habilitation figures in the field of Obstetrics and Gynecology were evaluated about gender distribution. All data were received from federal institutes.

**Results:**

A total of 46.7% (*n* = 95,234) of all inpatient doctors were female. A total of 46.7% (*n* = 95,234) of the physicians in hospitals were female. A total of 46% (1,832/3,958) were the portion of females as assistant physicians, 39.8% (*n* = 45.551) as specialists, 35.3% (*n* = 18789) as senior physicians, 25.1% (*n* = 2394) as first senior physicians and 25% (*n* = 10) as chief physicians in hospital. A total of 64.6% (*n* = 3958) of the physicians in Obstetrics and Gynecology were female. A total of 46% (1,832/3,958) were the portion of females as assistant physicians, 64.6% (*n* = 3958) as specialists, 65.0% (*n* = 1919) as senior physicians, 26.4% (*n* = 207) first senior physicians and 25% (*n* = 10) as chief physicians in Obstetrics and Gynecology.

**Discussion:**

The problem with the gender gap in medicine, does not seem to be access to teaching or starting a residency. But in the functions with increasing responsibility and management functions, e.g., as senior physicians, women are already rarely seen. In Obstetrics and Gynecology, too, there is a shortage of women in leading positions, despite the relatively high numbers, for example as senior physicians. Factors like maternity and establishing a family are points mentioned therefore, but also stereotypes seem to be considerable facts.

**Conclusion:**

However, it is important to recognize the need for more women in higher positions in medicine and actively work to encourage more women to choose a career in medicine.

## Introduction

The term “Gender Gap” refers to the gender-specific inequality that exists in many areas of society. Essentially, Gender Gap means that there are differences between genders that can affect various aspects of life, such as access to education, employment opportunities, wages, political representation, or healthcare (1). The Gender Gap can have both positive and negative impacts on society, depending on whether the differences between genders result in one group being disadvantaged or preferred. Eliminating the Gender Gap is an important goal of gender equality efforts to ensure that all people have equal rights and opportunities regardless of their gender (2). There is also a Gender Gap among physicians. This Gender Gap refers to differences between men and women in terms of access to medical education, career development, and leadership positions in medical practice and research. Although women now make up most medical school graduates in many countries, they are often underrepresented in higher positions (3). After medical school, physicians in Germany must complete a residency, which lasts 6 years for obstetrics and gynecology. During this time, the physicians are employed as assistant physicians. After completion of the residency, the physician is a specialist. In addition to the acquisition of the qualification, there are also hierarchical structures in the clinic, so a doctor can also become a senior physician in recognition of his performance and assumption of responsibilities. The qualification of specialist is not necessary for this, but often already exists. At the top of the hierarchy is the chief physician.

For example, there are fewer female chief physicians, professors, or senior female scientists in medical research. The reasons for this can be diverse, including gender biases, discrimination, and structural barriers such as inadequate support for work-life balance. Eliminating the Gender Gap among physicians is important to ensure that women are equally represented in medical practice and research, and to ensure that all patients, regardless of their gender, receive equal medical care. The consequences of this inequality are far-reaching and affect both healthcare and society (4, 5). It is therefore crucial that measures are taken to improve women’s access to medical education and career development and to promote gender equality in medicine. Although the Gender Gap in medicine varies in different specialties and countries, there are some areas that are particularly affected. One example is surgery, where women are often underrepresented, especially in surgical subspecialties such as cardiothoracic surgery or neurosurgery (6, 7). In other areas such as oncology, cardiology, and critical care medicine, women are also frequently underrepresented in higher positions and leadership roles (8–10). These differences can have negative effects on healthcare as women may not be able to benefit equally from certain specialties and treatment options. Therefore, it is important for the medical community to recognize the challenges of the Gender Gap in various specialties and take appropriate measures to ensure that women have equal career opportunities and access to medical care. Although gynecology is a specialty that is traditionally dominated by women, there is still a Gender Gap in this field especially in the leading positions. One challenge is that there are often few career opportunities in gynecology beyond clinical practice, which means that women are often underrepresented in higher positions such as chief physicians or professorships in gynecology (11). Furthermore, there are also challenges regarding the balance of family and career that can affect the career development of women in gynecology. For example, pregnancies and childcare can make it difficult for women to maintain or advance in higher positions. It is therefore important that the medical community is aware of the gender gap in gynecology and takes measures to ensure that women have equal career opportunities and that their health needs are adequately considered (12). The aim of this study is therefore to analyze the gender-specific development in the field of Obstetrics and Gynecology in Germany over the past 20 years and to survey the current *status quo*.

## Materials and methods

For the purpose of the study, an analysis was carried out on the development of female graduates of human medicine, the proportion of women in contract medical care and clinical care, as well as the gender-specific evaluation of obtaining a gynecological/obstetric additional qualification. Habilitation figures in the field of Obstetrics and Gynecology were evaluated about gender distribution. The hypothesis of the present study was that the number of female doctors in the field of Obstetrics and Gynecology is increasing, but there remains a significant imbalance in gender distribution.

### Ethics commission

The responsible Ethics Committee of the University of Jena was informed and did not raise any objections to the study (Reg. -Nr.:2019-1456-Bef).

### Data collection

Between January and April 2023, this study analyzed publicly available data on gender distribution in Obstetrics and Gynecology in Germany over the past 20 years from various national and international sources. These publicly available sources were:

– Statistical data on graduates of the human medicine program in Germany [Health Reporting of the Federal Government, (11)].
– Development in German hospitals [Information System of Health Reporting of the Federal Government, supported by the Robert Koch Institute (RKI) and the Federal Statistical Office, (12)].
– Development in outpatient care [Federal Physician Register of the National Association of Statutory Health Insurance Physicians, (13)].
– Data on the recognition of additional training in Obstetrics and Gynecology from 2016 to 2018 [German Medical Association, (13, 14)].
– Gender analysis of the leadership of a gynecological/obstetric university hospital (website of the 37 German university hospitals where the study of human medicine is state-approved).
– Data on successfully completed habilitations in the field of Obstetrics and Gynecology from 2002 to 2020 [University Statistics of the Federal Statistical Office, (15, 16)].

### Data analysis

Statistical analysis was performed using IBM SPSS Statistics 29.0. Nominally scaled data were analyzed using the chi-square test. Measured metric values were examined for significant differences with the Mann-Whitney-*U*-test. The significance level was set at the 5% level (*p* < 0.05). Charts were produced using Microsoft Excel (Excel 2019, Microsoft Corporation, Redmond, Washington, DC, USA).

## Results

### Medical education

**FIGURE 1:**
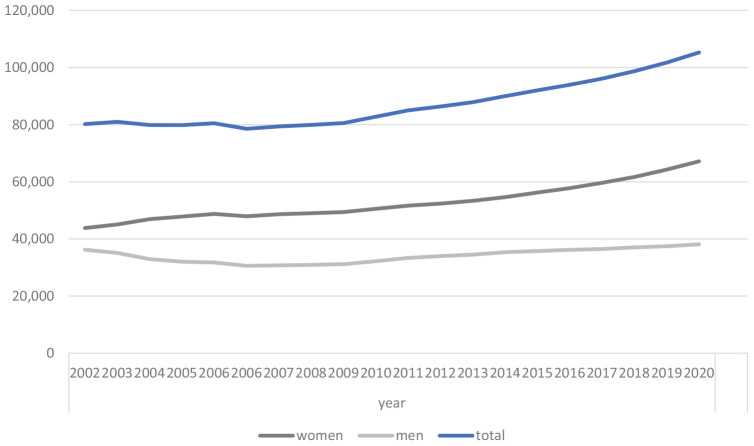
Medical students in Germany subdivided by gender over the years 2002–2020 in total amounts.

Currently, around 98,700 people are studying human medicine in Germany–of which approximately 63 percent are women (62,586/98,733). The number of first-year students has slightly increased over the past 10 years and decreased in 2021 compared to 2020. In 2021, around 1,750 more people were studying in the first semester than in 2012. The number of applicants for a medical degree program continues to far exceed the number of available spots. In 2021, 12,433 female and 6,337 male human medicine students completed their studies. Over the past 20 years, the number of female human medicine students has steadily increased from 43,807 in 2002 to 67,149 in 2020. This represents an increase of almost 53% (see Figure 1).

### Specialist training/residency

Currently, there are a total of 409,121 physicians working in Germany. Of these, 48.2% (*n* = 197,036) are female doctors, with 13,422 working as specialists in gynecology and obstetrics. In general, the proportion of physicians under the age of 35 has decreased from almost 25 to 19% in the last 25 years. The number of physicians who have been employed in an outpatient setting has increased by 660% in the last 25 years. In the field of gynecology and obstetrics, the highest proportion of practicing gynecologists and obstetricians are aged between 50 and 59 years (*n* = 6,609). The proportion of practicing physicians in the field of Obstetrics and Gynecology under the age of 34 is the lowest (*n* = 684). Epidemiologically, almost 60% of all specialists in Obstetrics and Gynecology are over 50 years old.

### Working place hospital

In 2021, a total of 203,286 male and female doctors were employed in the inpatient sector in Germany 46.7% (*n* = 95,234) of them were female.

A total of 43.7% (*n* = 88,803) were in specialist training, 56.0% (*n* = 49692) of them were female. Of these, 56.3% (*n* = 114,483) were male and female doctors with completed specialist training, 39.8% (*n* = 45.551) of them were female.

**FIGURE 2:**
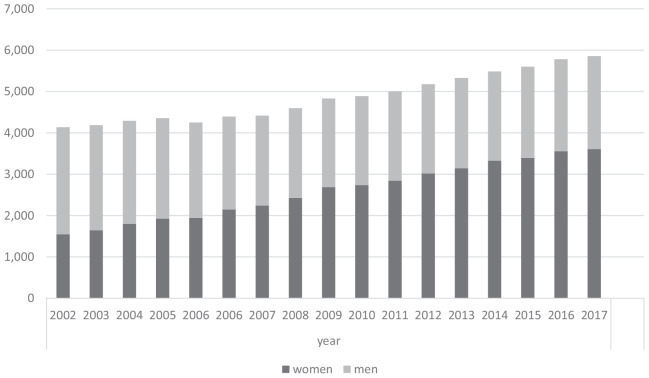
The development of the number of physicians of obstetrics and gynecology employed in the hospital from 2002 to 2017 in Germany is shown in figure.

In 2021, 26.2% (*n* = 53,283) of the hospital-employed doctors were working as senior physicians and 35.3% (*n* = 18789) of them were female. Of these, nearly 1 percent did not have completed specialist training. A total of 8.0% (*n* = 53.283) were employed as first senior doctors with leading functions. Of these, 99 percent had completed specialist training and in total 14.7% (*n* = 2394) of the first senior physicians were female. Around 5% are working as female chief physicians. A total of 133,731 male and female assistant doctors were employed in German hospitals in 2021. The development of the number of physicians employed in the hospital over time is shown in Figure 2.

A total of 3.0% (*n* = 6,127) of the specialists were employed as specialist doctors in Obstetrics and Gynecology in hospitals and 64.6% (*n* = 3958) were female. Also 65.0% (*n* = 1919) of senior physicians in Obstetrics and Gynecology (*n* = 2954) were female. But only 26.4% (*n* = 207) of first senior physicians in Obstetrics and Gynecology (*n* = 784) were female.

**FIGURE 3:**
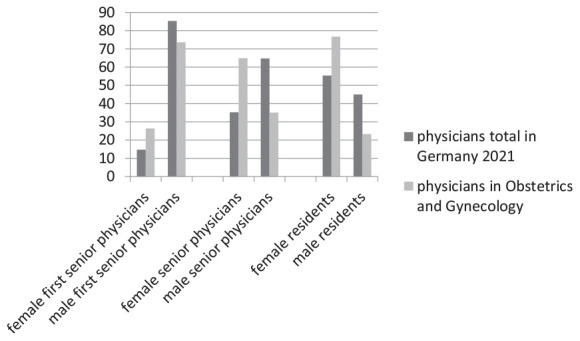
Proportion of genders in the positions: residents, senior physicians and first senior physicians in medicine and in obstetrics and gynecology in 2021 in Germany.

Compared to the total number of women or senior physicians working in hospitals, statistically more women are employed as senior physicians in the field of Obstetrics and Gynecology (*p* > 0.001). Out of the 95,234 female doctors working in hospitals, 74,060 (77.8%) are working as assistant physicians. Compared to the total number, roughly 46% (1,832/3,958) of women within gynecology are working as assistant physicians, while nearly 78% of female doctors working in hospitals are working as assistant physicians (Figure 3). Therefore, statistically more assistant physicians are working in other fields than in Obstetrics and Gynecology (*p* > 0.001).

### Specialists in obstetrics and gynecology

The number of specialist doctors in Obstetrics and Gynecology who work in a hospital setting has increased for both genders in the last 20 years, with the female proportion rising by almost 60% from 2002 to 2017 (from 1,546 to 3,612). In comparison to male specialists in gynecology, the proportion of female specialists in gynecology has shifted from being in the minority to being in the majority: in 2002, the number of female gynecologists was 1,546, and in 2017 it was 3,612. In 2021, 6,127 male and female specialist doctors in Obstetrics and Gynecology were employed in a hospital setting, of which 3,958 were female specialist doctors in Obstetrics and Gynecology working full-time.

### Leadership in gynecology/obstetrics at German universities

At the 39 state-run university hospitals in Germany, out of the 40 chief physician positions, 25% (*n* = 10) are held by women and 75% (*n* = 30) by men. Out of the 80 leading senior physician positions to be filled, 37.5% (*n* = 30) are held by women and 62.5% (*n* = 50) by men.

### Specialization

**FIGURE 4:**
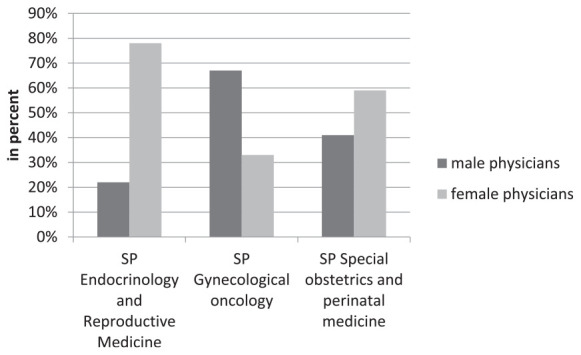
Additional designation of specialization in obstetrics and gynecology divided by gender.

A total of three specialized designations can be achieved in the field of Obstetrics and Gynecology: Endocrinology and Reproductive Medicine, Gynecologic Oncology, and Special Obstetrics and Perinatal Medicine. In 2020, a total of 1,157 doctors in the field of Obstetrics and Gynecology had a specialized designation: 18.9% (*n* = 219) in Endocrinology and Reproductive Medicine, 42.3% (*n* = 489) in Gynecologic Oncology, and 38.8% (*n* = 449) in Special Obstetrics and Perinatal Medicine. Of these 1,157 doctors, 52% (*n* = 599) were female and 48% (*n* = 558) were male with a specialized designation. Of the 219 doctors with the specialized designation in Endocrinology and Reproductive Medicine, 78% (*n* = 171) were female doctors of the 489 doctors with the specialized designation in Gynecologic Oncology, 33% (*n* = 161) were female doctors and of the 449 doctors with the specialized designation in Special Obstetrics and Perinatal Medicine, 59% (*n* = 267) were female doctors (Figure 4).

**FIGURE 5:**
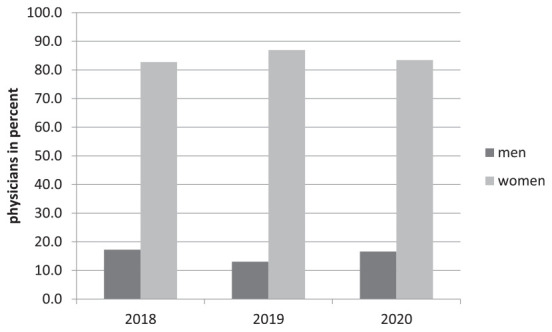
Physicians with specialization in obstetrics and gynecology 2018–2020 divided by gender.

In 2020, a total of 700 doctors achieved the specialist title in gynecology and obstetrics. Of these, 16% (*n* = 116) were men and 84% (*n* = 584) were women (Figure 5).

### Habilitations

**FIGURE 6:**
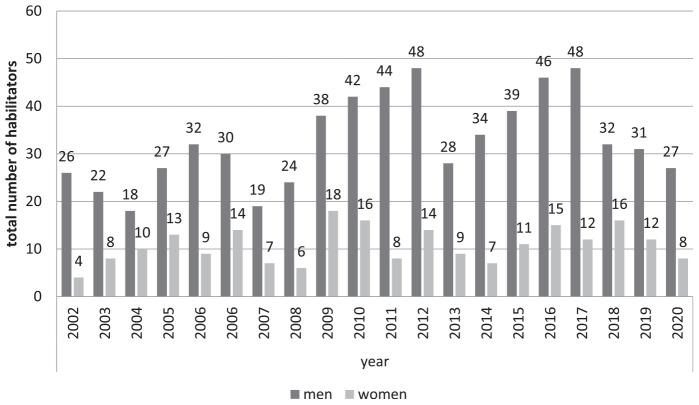
Habilitators in obstetrics and gynecology from 2002 to 2020 in Germany divided by gender.

From 2002 to 2020, a total of 872 doctors in Germany habilitated in the field of gynecology and obstetrics. Of these, 25% (*n* = 217) were women and 75% (*n* = 655) were men. The number of habilitations has decreased in recent years for both genders (Figure 6).

## Discussion

The gender gap in medicine has implications for many areas of healthcare (17). Here are some examples:

### Diagnosis and treatment

Studies have shown that men and women often have different symptoms for the same conditions. If doctors do not take these differences into account, they may make incorrect diagnoses or prescribe ineffective treatments (18).

### Research

Because women are often underrepresented in medical research, study results may not be applicable to both genders. This can lead to suboptimal treatments for women and impede understanding of certain diseases (19).

### Career opportunities

Women are often underrepresented in the medical industry and have fewer opportunities for leadership positions. This can result in women having less influence over decisions that impact patient health (20).

### Salaries

Women in medicine often earn less than their male counterparts. This can discourage women from pursuing a career in medicine or staying in the field (21).

These are just a few examples of how gender differences can impact healthcare. It is important to raise awareness of these issues and take action to ensure that women and men have equal opportunities and access to medical care (22). There is a growing number of female doctors in medicine.

In many countries, there are now more women than men who study medicine and work as doctors. This is a positive development as it increases diversity in the medical field and can contribute to better patient care. Women may approach certain aspects of healthcare differently than men, and their presence in the medical field can help better consider certain needs and perspectives (22). However, there are still some challenges that female doctors face, particularly about balancing family and career. In some medical specialties, women are still underrepresented, and there are still obstacles that can make it more difficult to pursue a successful career in medicine (23).

The problem with the gender gap in medicine, does not seem to be access to teaching, the majority of students are female. Starting a residency is not the problem either, again the majority is female. But by the time residency is completed, it no longer shows a majority of initially starting female physicians. In the functions in the hospital with increasing responsibility and management functions, e.g., as senior physicians, women are already rarely to be seen. Among first senior physicians and chiefs, women can be found in single digits. So it can be concluded that on the way to senior positions, women are not climbing the career ladder. But why is that? Morrison et al. in his recently published study for orthopedic surgeons, showed that maternity was a fact of being more difficult to advance in surgical specialties (24). This is an often cited problem that women have to take care of family and job is a point here (25).

In the study by Smith et al. it is also shown that the pandemic has had a further detrimental effect on women in leadership positions. thus, additional praying tasks have fallen on women. Women need up to 15 h per week to care for the household and children (26).

However, this does not seem to be the only factor. In particular, the stereotypes of the woman by herself, plays a major role. Thus, women more often go part-time and take jobs in medicine outside the hospital and often away from patient care (27).

Karaharju-Suvanto et al. showed gender differences in the career choices of dentists in his study. Women saw themselves as comforters, while men saw themselves more as technicians. Cultural ideals regarding appropriate occupations for men and women may have influenced their career choices. It appeared that male graduates seemed to concentrate in the private sector and chose more financially rewarding specialties that required technical skills, while women tended to prefer the public sector and focused on specialties that focused on social care and health promotion (28).

In the field of gynecology, there is a higher proportion of female doctors compared to other medical specialties. This is likely because gynecology deals with the health and wellbeing of women, and only few women prefer to be treated by a female doctor (29). According to a survey by the German Medical Association in 2020, about 69% of specialist doctors in Obstetrics and Gynecology are female, compared to an average female representation of about 46% among all specialist doctors. In other specialties such as surgery, orthopedics, or urology, the proportion of women is significantly lower (14).

In Obstetrics and Gynecology, too, there is a shortage of women in senior positions, despite the relatively high numbers, for example as senior physicians.

A greater number of women in this field could also help bring a more diverse perspective to medical practice and improve overall healthcare (30).

It is important to note that hiring doctors, whether male or female, depends primarily on their skills and qualifications, not their gender. The best person for the job should always be selected, regardless of gender, race, or other personal characteristics. Nevertheless, it is important to encourage women to choose a career in medicine and actively address the obstacles and barriers they may face. This can help create a fairer and more diverse medical profession that better considers the needs and perspectives of all patients and doctors (31). The gender of a physician has no impact on their abilities as a gynecologist. A gynecologist, whether male or female, should have the necessary expertise, experience, and empathy to care for women in all aspects of reproductive health. Both male and female gynecologists can provide excellent care, and it is important for women to choose the physician with whom they feel most comfortable and can trust. Trust and open communication between physician and patient are crucial for good medical care. There are an increasing proportion of women in healthcare professions such as nursing and medical care (23). This change has various impacts on healthcare:

### Improved patient care

Research has shown that female healthcare professionals often have a stronger ability to empathize and communicate with patients, leading to higher patient satisfaction and better outcomes.

### Gender pay gap

Although women make up the majority of workers in healthcare, they are often concentrated in lower-paid roles such as nursing, while men are more likely to be found in higher-paid positions such as medicine and surgery. This leads to a gender pay gap (32).

### Workload

As women often work in patient care and nursing, they often have a higher workload and must deal with stressful situations that can lead to burnout.

### Potential changes in healthcare policy

With women playing a greater role in healthcare, their interests and needs can be better represented in healthcare policy.

Overall, the higher amount of women in healthcare has both positive and negative impacts. While women can often provide better patient care, they must also deal with challenges such as the gender pay gap and burnout (31, 32). The disadvantages of the gender gap are certainly individual for women in terms of career development, financial consideration and personal health. But the facts are also negative from a societal perspective, as less income is available for the economic system and the number of illnesses and thus healthcare costs increase due to psychological challenges such as mobbing and burnout (33).

There is a shortage of women in the areas of research work and the end point of habilitation. Only a quarter of habilitators in Obstetrics and Gynecology are women, even though they make up a majority of physicians. Often this is an additional work and can usually no longer be done by women in the medical professions in their quitting time.

In Germany, the problem for women in obstetrics and gynecology does not seem to be access to education or even the further path to becoming a senior physician, but here, too, there are no women in management positions.

## Conclusion

The hiring of doctors should always be based on their skills, experience, and qualifications, not on their gender. However, it is important to recognize the need of women in medicine and actively work to encourage more women to choose a higher career in medicine.

This goal is difficult to achieve, it is essential to show the possibility of reconciling work and family, for example with kindergartens with appropriate opening times, breastfeeding options and flexible working hours. But that’s not enough, especially stereotype thinking in Germany in relation to the additional challenges that women have to meet must be reduced. Women should be given the opportunity to continue their career path without being pushed into other jobs or part-time, which often block managerial positions. Furthermore, the option of an academic career must also be supported, if necessary with mentoring programs, provided working hours, but above all networking. Only a rethinking of the women concerned and the men in decision-making positions can reduce the gender gap in managerial positions in obstetrics and gynecology.

## Data availability statement

The datasets presented in this study can be found in online repositories. The names of the repository/repositories and accession number(s) can be found in the article/supplementary material.

## Author contributions

SH, IG, ES, and FL contributed to the design and implementation of the research, analysis of the results, and the writing of the manuscript. NE analyzed the data, supervised the study, and edited the manuscript. MK performed the statistical analysis. All authors contributed to the article and approved the submitted version.
